# Treating sarcopenia: the LACE trial

**DOI:** 10.1002/jcsm.13004

**Published:** 2022-04-19

**Authors:** Andrew L. Clark, Alexandra A.I. Abel

**Affiliations:** ^1^ Department of Academic Cardiology, Hull and East Yorkshire Medical Research and Teaching Centre Castle Hill Hospital Kingston Upon Hull Cottingham UK

## Background

As the population ages and as chronic disease becomes something that patients live with, rather than die from, the problem of frailty is rapidly becoming a scourge. Although we all recognize frailty when we see it, trying to define it can be difficult: different definitions of frailty can lead to large differences in its apparent point prevalence.^1^ Nevertheless, a key component of frailty is loss of muscle mass or sarcopenia.^2^ Sarcopenia is associated with a huge variety of adverse outcomes, ranging from increased risk of fractures, greater risk of hospitalization, and higher risk of death. Over the last 10 years or so, as readers of this journal will know well, there have been intense efforts to understand the pathophysiology of sarcopenia, its clinical consequences, and, crucially, whether it might be treatable.

In patients with chronic heart failure, cachexia was first systematically investigated in the late 1990s.^3^ Loss of muscle bulk is a common feature^4^, ^5^, ^6^, ^7^ and strongly related to an adverse outcome.^8^ One of the great triumphs of modern medicine has been the pharmacological therapy of chronic heart failure in which blockade of neurohormonal activation dramatically extends both quality and length of life.^9^ Intriguingly, one of the effects of successful treatment is to prevent weight loss and to enhance weight gain, an effect seen with both angiotensin converting enzyme inhibitors (ACEi)^10^ and beta adrenoceptor antagonists (β‐blockers).^11^

Another possible approach to the management of sarcopenia might be dietary supplementation. Leucine, an essential amino acid, might have beneficial effects on sarcopenia. Leucine is one of the three essential branched‐chain amino acids (valine, leucine, and isoleucine). All three are associated with enhanced protein synthesis.^12^ A meta‐analysis of the available data suggests that leucine supplementation leads to an increase in lean muscle mass in people with sarcopenia.^13^

## The LACE trial

It is in this context that we read the results of the LACE trial, published in the current issue.^14^ The LACE investigators randomized 145 subjects with sarcopenia to receive an ACEi, perindopril, or placebo; and leucine or placebo, using a 2 × 2 factorial design. The primary end‐point was change in the short physical performance battery (SSPB) score over 1 year: the score assesses balance, time taken to stand from a chair 5 times, and walk speed over 4 m.^15^

In the event, there was no effect of either intervention on the SSPB score, even when adjusted for differences in baseline covariates. There were no effects on any of the secondary end‐points, including quadriceps strength, muscle mass, or 6 min walk test distance. Whilst leucine was well tolerated, perindopril was associated with more adverse events and a lower quality of life. There was no interaction between perindopril and leucine.

## Why was the trial neutral?

Another neutral trial of treatment when a properly conducted, randomized, double‐blinded study is carried out following the enthusiasm generated by earlier, perhaps less rigorously conducted, studies. Why do two apparently promising treatments have so little effect?

It may be that the patients were not suffering from severe sarcopenia and hence were not very likely to respond to treatment. There were only two deaths, a small number for patients with sarcopenia. Many of the patients did not fulfil the diagnostic criteria for sarcopenia, perhaps because the authors used bioimpedance measures of muscle mass as an inclusion criterion, not dual energy X‐ray absorbtiometry. In support of this notion, a *post hoc* analysis suggested that there might have been a beneficial effect of leucine in patients meeting the 2019 definition of sarcopenia, although this finding must be treated with great caution.

The lack of effect of perindopril may not be surprising. ACEi have effects in people with heart failure in whom activation of the renin‐angiotensin system is a prominent feature but might not be effective in people with other causes of sarcopenia. The doses used for the interventions may simply have been inadequate in patients with sarcopenia. Leucine might not have the effects seen in *in vivo* and animal studies in intact humans, particularly those with advanced sarcopenia. The interrelations between diet and muscle bulk are more complex than might be solved by supplementation with a single nutrient, and for leucine to work might require, for example, a generalized increase in protein intake or an additional micronutrient supplementation.^16^ In addition, an increase in muscle bulk might only be seen in those undertaking addition exercise training.

Most disappointing to the investigators was the low recruitment rate. LACE was originally planned to have 440 participants, but only a third of that number was recruited. In addition, there was a 25% drop‐out rate by the time of the 1 year follow up visit. A recurring problem with research into sarcopenia is that it is a condition overwhelming affecting older people, many of whom are no longer independent, many of whom have multiple comorbidities, and many of whom have some element of cognitive decline. For such people, the burden of entering a clinical trial with all its processes, paperwork, and follow‐up visits is simply insuperable. The process of conducting any trial thus paradoxically excludes from it those patients most likely to benefit from any intervention.

## Conclusions

The investigators are hugely to be congratulated on completing what was clearly a labour of love. LACE adds to our understanding of the possible treatment of sarcopenia, but a major benefit is to highlight weaknesses in the current paradigm for conducting clinical trials in some conditions. Age‐related decline in function in all body systems is unlikely to respond to simple interventions: a complex underlying physiological process will require a complex intervention to reverse it. Some combination of exercise, cognitive support, dietary supplementation, and even pharmacological intervention might eventually be helpful in sarcopenia, but we may have to change the way we approach clinical trial design in an elderly and frail population: a simplified consent process, not requiring pages of documentation, and simple endpoints, not requiring hospital visits, may be the way to go.

## Conflict of interest

There are no conflicts of interest to declare.
